# Genetic Mapping of the 22q11.2 Deletion Syndrome (DiGeorge Syndrome) Microdeletion Types Revealed Novel Candidate Breakpoints

**DOI:** 10.3390/genes17020248

**Published:** 2026-02-22

**Authors:** Louis Papageorgiou, Elena Nikolopoulou, Eleni Koniari, Kyriaki Hatziagapiou, Dimitrios Chaniotis, Apostolos Beloukas, George P. Chrousos, Elias Eliopoulos, Trias Thireou

**Affiliations:** 1 Department of Biomedical Sciences, University of West Attica, Agioy Spyridonos 28, 12243 Egaleo, Greece; 2 Laboratory of Genetics, Department of Biotechnology, Agricultural University of Athens, 11855 Athens, Greece; 3 University Research Institute of Maternal and Child Health and Precision Medicine, School of Medicine, National Kapodistrian University of Athens, 11527 Athens, Greece; 4 Clinical and Translational Research Endocrine Unit, School of Medicine, National Kapodistrian University of Athens, 11528 Athens, Greece

**Keywords:** 22q11.2 deletion syndrome, DiGeorge Syndrome, genomics, clinical genomics, genetics, LCRs, palindromic analysis, break points

## Abstract

**Background**: 22q11.2 deletion syndrome (DiGeorge Syndrome) is a rare disorder that involves a de novo hemizygous microdeletion within the 22q11.2 chromosomal locus. Individuals affected by this condition display a wide array of clinical phenotypes as well as haplotype sequences, which render understanding the genotype–phenotype relationship quite difficult. Additionally, the complex structure of the 22q11.2 low-copy repeats (LCRs), which usually inhibits sequencing efforts, has complicated the study of possible breakpoints that instigate the deletion events. In this study, 22q11.2 deletion syndrome is investigated on a genomic and phenotypic level for the purpose of determining the impact of each deletion type and identifying possible candidate breakpoints. **Methods**: In the present study, a systematic review combined with a secondary genomic analysis has been executed following PRISMA guidelines using PubMed and Scopus publications in order to estimate its holistic genomic map, genomic functional elements, and key genomic regions such as LCRs. A statistical content analysis of the affected chromosomal regions was also performed. Groups of functional elements with common traits were composed, and their contribution to the deletion events was investigated. Finally, the 22q11.2 repeat regions were screened for palindromic AT-rich repeats. **Results**: Of the 8202 unique publications studied in this work, only 65 met the inclusion criteria. The estimated genomic map of 22q11.2 deletion syndrome in the secondary genomic analysis revealed 11 distinct microdeletions occurring between eight LCRs, and a new repeat region within the CES region (CESRR), of which the LCR22A-LCR22D deletion was the most frequently reported. Last but not least, the palindromic analyses indicated eight critical groups as candidate breakpoints that potentially form four distinct patterns, and ten palindromic AT-rich repeat (PATRR) regions were identified amongst LCR22A, LCR22B, LCR22D, LCR22F and LCR22H. **Conclusions**: The study results validate the differentiating clinical contribution between the proximal and the distal segments. Eight novel candidate breakpoints and five new PATRRs were identified that require further study to establish their involvement in 22q11.2 microdeletion events.

## 1. Introduction

### 1.1. Pieces of a Puzzle—A Brief History of 22q11.2 Deletion Syndrome

In 1955, a phoniatrist by the name Eva Sedláčková, whilst attending to her patients, noticed that a group of 28 children with hypernasal speech, reduced facial animation and atypical facial features exhibited congenital soft palate malformations, velopharyngeal insufficiency and some other developmental anomalies in different parts of their body, such as abnormalities in the fingers and toes or even congenital heart disease. By 1967, the number of patients had increased to 48. After consideration, Dr. Sedláčková theorized that the root cause of these manifestations might be found in the early stages of embryonic development [1]. Meanwhile, in 1959, D.H Lobdell noted a connection between congenital thymic aplasia with congenital hypoparathyroidism and hypocalcemia [2,3]. This association was confirmed again in 1965, by Dr. A. DiGeorge upon performing an autopsy on three infants and later in 1968 after he and Dr. Kirkpatrick conducted another case study of infant patients [4]. They also concluded that the congenital absences of the thymus and parathyroid glands can be accompanied by related manifestations, such as anomalies of the aortic arch [5]. Thus, DiGeorge Syndrome (DGS) was established. Around the same time, W. Strong et al., upon examining a family whose members exhibited distinctive phenotypes, suggested that the “unknown” syndrome of both physical and cognitive disorders he had encountered followed a dominant-inheritance pattern [6]. A year later, G. Cayler conducted his own case study of patients with cardiofacial anomalies and noted several chromosomal aberrations [7]. Across the globe, similar phenotypes began to be recorded in Japanese literature in the mid-70s by physicians Dr. Kinouchi and Dr. Takao, who coined the term Conotruncal anomaly face syndrome or Takao Syndrome [2,8]. In 1978, Shprintzen et al., after studying a group of children with cardiac, velopharyngeal and facial manifestations, as well as learning disabilities and speech and language impairments, described Velocardiofacial Syndrome (VCFS) [9]. Finally, De la Chapelle et al., in 1981, linked DiGeorge Syndrome to a deletion located in the 22q11 region of the 22nd chromosome [10]. By that point, a clearer picture was starting to form with each small piece of information. In 1993, Wilson et al. integrated DGS into the “new” CATCH22 Syndrome (Cardiac defects, Abnormal facies, Thymic hypoplasia, Cleft palate, Hypocalcemia), as it was considered to be part of the CATCH22 spectrum [11]. A breakthrough was made in 2005, when Shprintzen & Robin stated that all the previously described syndromes constitute a single condition that is now known as 22q11.2 deletion syndrome [12]. Looking at its rich history, it is not hard to understand why the same condition is attributed numerous aliases and diagnoses. With the aim of improving our understanding of this syndrome, the 22q11.2 locus continues to be studied on a genomic level through various mapping and genetic analysis efforts, as there is still much left unknown about the root cause and influencing factors that determine the genotype–phenotype relationship. While personalized medicine emerges as the practice of the future, understanding the dynamics of this relationship is crucial to successfully provide individualized medical care to patients with conditions on a broad and heterogeneous clinical spectrum.

### 1.2. The 22q11.2 Microdeletion—A Genetic and Phenotypic Review

22q11.2 deletion syndrome is the result of non-allelic homologous recombination (NAHR) events between chromosomes 22, which lead to the hemizygous microdeletion of the 22q11.2 region [13]. This mutation occurs mainly (90% of cases) *de novo*, although in some cases an autosomal dominant inheritance pattern has also been reported [13,14,15]. It has been estimated that this syndrome appears with a frequency of 1:4000 births; however, it has been suggested that this number could be between 1:2000 and 1:6395, while variations between ethnic groups are also noted [13,15]. The microdeletion most commonly extends to 3 Mb followed by 2 Mb and 1.5 Mb [14,16].

The clinical phenotype of 22q11.2 deletion syndrome is highly diverse and affects multiple organs due to the absence of a specific group of genes and other genomic functional elements [17,18]. The first clinical signs detected in infancy are associated with cardiac anomalies including Tetralogy of Fallot and interrupted aortic arch [19]. Patients with 22q11.2 deletion syndrome typically have craniofacial malformations, including characteristic features like hooded eyelids, a tubular nose, and low-set/dysmorphic ears [19]. Due to the underdevelopment of the parathyroid glands, several patients experience hypocalcemia and hypoparathyroidism, which can manifest as neonatal seizures. Furthermore, thymic hypoplasia leads to varying degrees of T-cell deficiency, increasing susceptibility to recurrent infections. Other common symptoms include neuropsychiatric disorders such as schizophrenia in adulthood, and immunodeficiency, which can lead to the emergence of related conditions such as autoimmune disease and atopy or increase the risk of infection [13,15,20,21].

### 1.3. Low-Copy Repeats in the 22q11.2 Region and Their Breakpoint Sequences

Low-copy repeats (LCRs) are complex sequences that consist of repeating DNA subunits larger than 1 Kb in size and of high sequence identity (97–98%) [22]. These repeating subunits can form different patterns, with various numbers of copy repeats and even inverted configurations. This is further complicated by the fact that these subunits are subject to sequence variation amongst ethnic groups [23]. LCRs contain elements that are susceptible to rearrangements, known as breakpoints, which are responsible for the emergence of the microdeletion [22,23].

There have been eight, in total, identified LCRs in the 22q11.2 region (LCR22s), which have been listed alphabetically as LCR22-A through -H, respectively. Based on which of these LCR22s are involved, 22q11.2 deletion syndrome can be further categorized as proximal (sites LCR22A-D), central (sites LCR22B-D) and distal (sites LCR22C-H) [24]. Distal deletions have been attributed with milder phenotypes, whilst the proximal region LCR22A-B has been suggested to house the main contributing genes for palate anomalies and congenital heart disease [16]. Even though the 22q11.2 microdeletions mainly revolve around these LCR22s, in some cases atypical microdeletions have been reported that involve regions outside of the LCR22 sites or even single genes. Another region that seems to be implicated in some types of the microdeletion is the Cat Eye Syndrome region (CES) that is located before the LCR22A [22].

### 1.4. Diagnosis and Treatment

As mentioned above, there are certain characteristic symptoms for this syndrome, such as craniofacial anomalies, congenital heart disease, parathyroid and thymic hypoplasia, etc., which can provide great insight into the condition of the patient. A definite diagnosis, however, requires a genetic confirmation of the deletion, more specifically, to detect via genetic testing the absence of the 22q11.2 locus [13]. The most popular analysis tools for the detection of the deletion are FISH (Fluorescence In Situ Hybridization), MLPA (Multiplex Ligation-dependent Probe Amplification), Microarray SNP (Single Nucleotide Polymorphism Microarray), CGH Microarray (Comparative Genomic Hybridization Microarray) and qPCR, while sequencing techniques such as WES (Whole-Exome Sequencing) and WGS (Whole-Genome Sequencing) are also gaining traction [13,14,15]. Another potential method is Cell-free DNA testing, which, even though it has been noted to detect the deletion, is still under scrutiny to determine its success and reliability [14,25]. Finally, OGM (Optical Genome Mapping) has successfully detected the 22q11.2 microdeletion and could provide a non-invasive alternative means of genetic testing for prenatal screening [26].

Unfortunately, to this day, there is no cure for 22q11.2 deletion syndrome. Treatment essentially boils down to the management of the patient’s manifestations and frequent check-ups to monitor the progression of their symptoms. The ultimate goal is to achieve the highest level of physical, emotional and mental functionality by enlisting the help of multiple experts. A few ways to address the most common physical manifestations are corrective surgery to mend anatomical malformations, immunoprotective measures such as non-live vaccinations, prophylactic antibiotic or IVIG (intravenous immunoglobulin) care, vitamin D and calcium supplementation and even thymus transplant, which, although successful, might in some cases cause further complications. The mental and psychological aspects can be addressed through various practices such as speech therapy, psychotherapy and psychiatric support [13,15].

The present study aims to examine 22q11.2 deletion syndrome on a genomic and genetic level through the available scientific literature and information on its functional genomic elements from several biological databases. More specifically, data concerning microdeletion genetic locus/types and repeats such as LCRs are collected in order to pinpoint genomic loci of significance and to study the clinical consequences of each microdeletion molecular type. Our goal is the furtherance of existing information and scientific research regarding this syndrome in the hopes of aiding innovation in its genetic mapping, diagnosis, treatment and personalized medicine.

## 2. Materials and Methods

### 2.1. Dataset Collection and Pre-Analysis

The first part of the methodology involved a systematic review of publications describing this syndrome in order to collect sensitive information regarding the recorded microdeletion types and related information. The data utilized for the purposes of the present study were sourced from publications with English text, dating from 2012 to 2025, that are included in the databases PubMed and SCOPUS. Several different terms were used to create the initial dataset based on separate searches in the bibliographic databases, including “DiGeorge Syndrome”, “22q11.2 deletion Syndrome”, “LCRs in 22q11.2 region”, and “CES in 22q11.2 region” [27,28]. The selected publications were filtered by using inclusion and exclusion criteria as described in the PRISMA flow diagram (Supplementary Figure S1). Primarily, duplicate publications were removed from the general dataset by using the title information. Subsequently, all publications that did not have “title” or “abstract” information or were not written in English were removed from the dataset. Additional exclusion criteria were then applied, such as filtering out publications that did not correspond to a description of genetic or genomic data. Finally, publications that corresponded to syndrome “microdeletion types” or that identified the genetic locus of the “LCRs” were isolated. All extracted publications were evaluated manually for the information they described, and thus the final dataset studied was created (Supplementary Dataset S1) [29].

### 2.2. Determining the 22q11.2 Deletion Syndrome Microdeletion Types

22q11.2 deletion syndrome has been linked to more than one haplotype (microdeletion type), as different regions of varying extent and position have been found absent in patients diagnosed with this condition. In this study, the 22q11.2 deletion syndrome microdeletion types were determined through several studies that have been extracted from the previous step [16,22,30,31,32,33,34]. This collection includes clinical data from 66 patients in total and several 22q11.2 deletion syndrome reviews and metanalyses. It is important to note that neither is the extent of each existing microdeletion type well documented nor are the literature data in agreement regarding the exact genetic locus of the LCRs and the intermediate regions (IRs). The most extensively recorded molecular types are microdeletions A-D (3 Mb), A-C (2 Mb) and A-B (1.5 Mb); however, there is little information on distal deletion types. Thus, for the purposes of this study, the genetic locus, size and content of each repeat’s region was determined anew (Supplementary Dataset S2) [22,35,36,37,38,39,40,41,42,43,44,45,46,47,48,49,50,51,52,53,54,55,56,57].

### 2.3. Identification of Repeat Genetic Locus

In this step of the methodology, a genomic study was conducted in order to have a complete mapping of the syndrome based on the distinct regions of interest, the LCRs. The LCRs of the 22q11.2 locus have maintained a consistent presence on the human genome and are, therefore, a good reference point to determine the genetic locus of each microdeletion subtype. As a starting point, the genetic locus of the repeats LCR22A, LCR22B, LCR22C and LCR22D were determined based on the Vervoort and Vermeesch study (2022) [22]. The selected coordinates correspond to the T2T-CHM13v2.0 (24 January 2022) version of the human genome, as it is the most recent version without gaps in the 22q11.2 region [58]. The remaining repeats (CES repeat region and LCR22E-LCR22H) were determined by identifying regions rich in segmental duplications in the UCSC Genome Browser (UCSC Genome Browser on Human Jan. 2022 (T2T CHM13v2.0/hs1) (hs1)—Variation and Repeats) [58]. In order to evaluate the success and reliability of this method, the locations of LCR22A-LCR22D were similarly determined and compared to the sourced coordinates. The deviation wvas calculated as the difference between the approximate and the referenced coordinates and was depicted as (+) for surplus and as (-) for deficit in bases (b). This deviation was also expressed as a percentage (%) of the total length of the corresponding repeat region. Finally, the determined genetic locus for each repeat was cross-referenced with depictions of the 22q11.2 locus in several studies by examining the noted coordinates, neighboring genomic loci and size descriptions (Supplementary Dataset S3) [16,22,30,31,32,33,34].

### 2.4. Phenotype and Microdeletion Type Determination in 22q11.2 Deletion Syndrome

**Figure 1:**
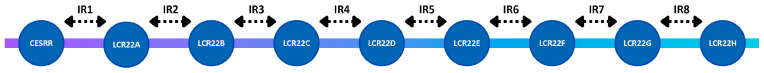
Visualization of the 22q11.2 deletion syndrome region based on the segment interest. In the figure are shown the 17 segments that have been studied, including CES, LCR22A-LCR22H and IR1–IR8.

The second part of the genomic analysis concerned the correlation of the identified microdeletions with candidate pathogenesis. To achieve this, every microdeletion molecular type was evaluated in terms of possible pathogenesis. For the purposes of this work, the deletion region of the syndrome (based on the various types of microdeletions) was divided into 17 segments, including CES, LCR22A-LCR22H and intermediate regions (IRs) 1–8 (Figure 1). Every element included in each segment of interest in the 22q11.2 region was examined for all associated clinical phenotypes listed on the ClinVar database, which are classified as pathogenic and likely pathogenic [59,60,61]. The results were catalogued and filtered for any duplicates, which were then removed. All 22q11.2 segments were matched to a collection of clinical phenotypes, and a pathogenesis map for the 22q11.2 region was formed. These results were compared to the documented diagnoses from several other studies [30,31]. More specifically, for each microdeletion type, the recorded clinical diagnoses were compared to the predicted phenotypes according to the pathogenesis map, and, if any matches were identified, they were noted (Supplementary Dataset S4).

### 2.5. Mapping of Genomic Functional Elements in the 22q11.2 Syndrome Microdeletion Types Based on Segment of Interest

The third part of the genomic analysis concerned the mapping of genomic functional elements in the 22q11.2 syndrome microdeletion types. A genomic map was constructed that was enriched with all the loci contained in the 22q11.2 region, in accordance with the T2T-CHM13v2.0 (24 January 2022) version of the human genome [58]. More specifically, the map concerns the area flanked by the CESRR and the LCR22H, which spans across the chr22: 15,709,205–25,164,881 region and includes 475 genomic elements. Firstly, the composition of the 22q11.2 region was examined by cataloguing the number and type (protein coding, non-coding, pseudogenes, miRNA, snRNA, snoRNA and immunoglobulin gene) of each functional element that comprises each microdeletion type, segment and identified repeat regions (Supplementary Dataset S5) [62]. The bounds of each segment, which are defined as the area flanked by repeated regions or microdeletion types, were set as the starting point of the leading and the end of the last repeat region. The degree of similarity between segments and repeat regions was also evaluated by identifying groups of recurring functional elements that are characterized by common traits such as family, function or structural components. Moreover, the number of segments that the functional elements of each group occupy was determined, and the sum of functional elements that are included in each group was calculated. Non-characterized loci were not included in this analysis. The groups were formed in accordance with the National Centre of Biotechnology Information (NCBI) and the HUGO Gene Nomenclature Committee (HGNC) [63]. The identified similarities were studied, with the aim to pinpoint functional elements of interest that could be possibly implicated in chromosomal rearrangements. The FAM230 non-coding elements and BCR pseudogenes have already been recognized as candidate causative elements in 22q11.2 deletion events [22,53,64]. As such, all types were examined with the purpose of identifying the ones which exhibit similar characteristics to FAM230 and BCRPs, namely (a) their prevalence in the 22q11.2 locus, (b) their relative position to the repeat regions and (c) the number and dispersion of functional elements they include (Supplementary Dataset S5). Moreover, as the A-D microdeletion is the most frequent of all documented microdeletions by a significant margin, it could be reasonably hypothesized that these regions are the densest in breakpoint sequences. Under this premise, a search was conducted in order to identify groups that are present exclusively in LCR22A and LCR22D [22]. The identified functional elements of interest serve as mere propositions for candidate breakpoints and require further evaluation and study, in order to determine whether they contain sequences susceptible to NAHR events or otherwise contribute to the 22q11.2 microdeletion events [22,53].

### 2.6. Palindromic AT-Rich Repeat Analysis of Repeat Regions in 22q11.2 Genetic Locus

Palindromic AT-rich repeats (PATRRs) have not only been linked to recombination events, but also to an increased likelihood of double-strand breakages as well [65]. Several repeats have already been mapped within the 22q11.2 locus, possibly implicating them in 22q11.2 syndrome deletion events. The final part of the genomic analysis involved identifying PATRRs in each repeat region by using Palindrome Analyser [66]. The FASTA sequences (T2T-CHM13v2.0) of the identified repeat regions were processed and examined for palindromic AT-rich repeats sized from 5 to 30 bases long, with an active filter for AT-repeats [66]. The repeats that exhibited a considerably high density were noted, and their respective coordinates on the human genome were ascertained. In order to determine other elements of interest regarding chromosomal rearrangements, a search for other distinct patterns was conducted. For the purposes of this study, patterns were defined as a procession of different functional elements occurring more than once in the 22q11.2 region in similar or inverted configurations [67,68,69]. These elements correspond to different characteristic groups that were defined during content analysis based on common traits. The patterns were considered as candidate breakpoints when located within or adjacent to repeat regions [68,70].

## 3. Results

### 3.1. The Dataset

The results of the systematic review showed that the earliest descriptions of the syndrome date back to the middle of the 20th century and mostly consider a variety of clinical manifestations and inheritance patterns, until 1969 when chromosomal abnormalities were first observed in patients. However, as this study focuses primarily on the genomic level, information regarding the hallmark manifestations, microdeletion variations and mapping of the 22q11.2 locus is annotated from sources that were published within the last decade. The total number of unique publications regarding 22q11.2 deletion syndrome, dating from 2012 to 2025, that were retrieved from PubMed and SCOPUS for several keyword searches amounted to 8202. As described in the PRISMA flowchart (Supplementary Figure S1), after the filtering process and manual evaluation, a collection of 65 publications was compiled, ten of which identified 11 distinct microdeletion types, while 55 offered insight into the genomic content of the 22q11.2 locus and reported repeats’ genetic loci or affected genes.

### 3.2. The 22q11.2 Deletion Syndrome Microdeletion Types and Their Features

**Figure 2:**
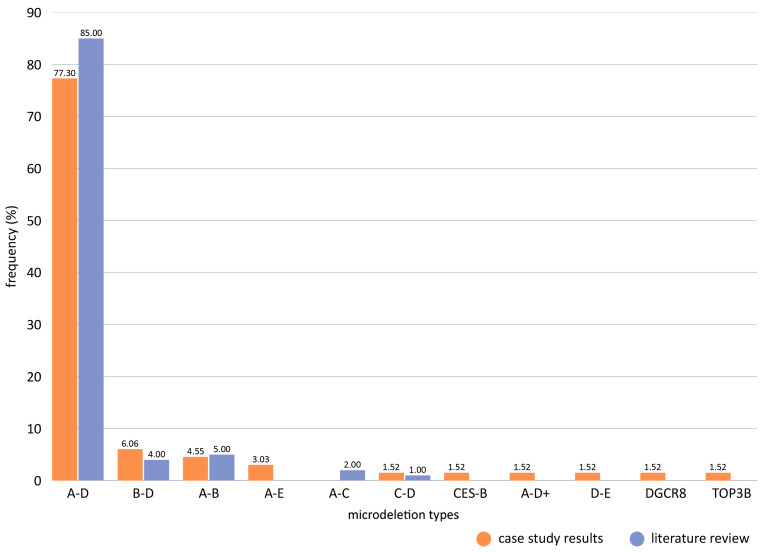
Occurrence of the 22q11.2 deletion syndrome microdeletion types. A statistical analysis of microdeletion type occurrence in 22q11.2 deletion syndrome patients and a visual comparison of bibliographical and case study-related results. This comparison is meant to be illustrative rather than representative.

A total of eleven microdeletion types were identified in publications, which included both genetic reviews and case studies. Often, the main focus in 22q11.2 syndrome reviews is the proximal (LCR22A-LCR22D) section of the genetic locus, so naturally most microdeletions they included correspond to this region. During research for the present study, the subtypes for A-D microdeletion (85%), A-B microdeletion (5%), B-D microdeletion (4%), A-C microdeletion (2%) and C-D microdeletion (1%) were catalogued, along with their corresponding frequency (Figure 2).

The most dominant molecular type is the A-D microdeletion, as it exhibits the highest frequency in both documented patient statistics and the case study sample (77.30–85%) (Supplementary Dataset S2). The initial findings of the genomic study showed that, in terms of size, the largest of the documented microdeletions was determined to be the CES-B microdeletion, which was approximated to span 5.1 Mb, but its size could not be confirmed in the available bibliography (Table 1) [16,30]. The A-D+ deletion’s exact size cannot be calculated since the precise limit it extends to is not disclosed by the publishers of the case study (Table 1). However, it can be safely surmised that its approximate size slightly exceeds that of the A-D microdeletion, which reaches about 3 Mb in length. The smallest microdeletions documented in the case studies are the single-gene deletions *DGCR8* (31,771 b) and *TOP3B* (25,765 b), which, although minute, were sufficient to produce recognizable manifestations of 22q11.2 deletion syndrome [30,71,72,73]. Overall, the calculated length of the microdeletion types, is found to be in agreement with the current literature that has been used. The microdeletions A-D, A-C, A-B, B-D and C-D are reported to extend 3 Mb, 2Mb, 1.5 Mb, 1.5 Mb and 1 Mb respectively, further supporting the presented results, notwithstanding a deviation of 0.1–0.5 Mb (Table 1) [30].

### 3.3. The Identified Repeats in 22q11.2 Deletion Syndrome

The positions of the proximal LCR22s (LCR22A, LCR22B, LCR22C and LCR22D) were referenced from the literature [22], and their genetic loci in accordance with the T2T-CHM13v2.0 version of the human genome were estimated in this step of the genomic analysis (Table 2). The same positions were also identified by screening each of the same four LCR22s for segmental duplications in the UCSC Genome Browser [74]. The deviation in size between the screening results and the referenced coordinates was satisfactorily small (−0.15% to 5.38%); therefore, the LCR22 identification method has been deemed successful. Thus, utilizing the aforementioned method, the remaining repeat positions (CES candidate repeats and LCR22E through LCR22H) were estimated (Table 2). The largest repeat region is found within the CES region and spans about 2Mb, while LCR22E is the smallest in size, extending 35 Kb, which is in agreement with the findings of Shaikh et al. 2007 [75]. Shaikh et al. 2007 also estimate that LCR22F spans more than 370 Kb, which aligns with the screening results, as LCR22F is calculated to be 397.7 Kb long [75]. There is little available information regarding the size and location of the LCR22s in the current scientific literature, which can be partly attributed to the limitations of assembly efforts regarding regions of high sequence identity and structural variation. Hitherto, it has been well established that the eight LCR22s (proximal LCR22A-D and distal LCR22E-H) are implicated in 22q11.2 deletion syndrome, and the involvement of an additional element within the CES region has also been proposed; however, the precise coordinates, sequences and breakpoints have not yet been described with certainty [76]. Similarly, the findings of the present study are mere approximations that aim to offer insight into the genotype–phenotype relationship in 22q11.2 deletion syndrome.

### 3.4. Phenotype and Subtype Determination of 22q11.2 Deletion Syndrome

22q11.2 deletion syndrome is related to an extensive array of clinical phenotypes that involve many different body systems, contributing to the high degree of heterogeny observed amongst patients affected with this condition [77,78,79,80]. In this part of the genomic analysis, the genotype–phenotype relationship was investigated by inspecting the documented microdeletions and their corresponding manifestations in each case study, in order to pinpoint a possible linkage either to the extent or the location of the deletion [81]. No such link was observed, as either the diagnoses varied greatly amongst patients with the same microdeletion type or the available data was insufficient, which could allude to the involvement of epigenetic factors or perhaps modifying elements. In terms of phenotypic density, namely the total related clinical phenotypes correlated with all functional elements of a segment, a high number of related diseases was observed between IR1 and IR5. For the purpose of studying the genotype–phenotype correlation in 22q11.2 deletion syndrome, a pathogenesis map was constructed that showcases all correlated clinical phenotypes for each functional element of the chr22: 15,709,205–25,164,881 region (Supplementary Dataset S4). The most prevalent proved to be the phenotypes for Cat Eye Syndrome (CES), Chromosome 22q11.2 microduplication syndrome (17 segments each) and schizophrenia (16 segments), while also a significant range is occupied by the phenotypes for intellectual disability/mild intellectual disability (13 segments), epilepsy, global developmental delay (12 segments each) and DiGeorge Syndrome (11 segments) (Table 3).

The IR2 area was identified as the most clinical phenotype-rich region, including 71 distinct phenotypes, followed by LCR22A (50 phenotypes) and LCR22D (49 phenotypes). Notably, the region LCR22A-LCR22D exhibits considerable density, which can be possibly attributed to the fact that this region is generally more thoroughly researched. The success of the map to reliably prognosticate the pathogenetic scope of each 22q11.2 deletion subtype was moderate. Whilst some of the reported diagnoses were successfully prognosticated, some clinical phenotypes, though exhibited by the patient, were expected to be linked with another segment of the 22q11.2, beyond the bounds of the corresponding deletion subtype (Table 3). A couple of the limitations need to be acknowledged, namely the limited sample of atypical deletion case studies and insufficient genotypic and phenotypic data.

### 3.5. Mapping of Functional Genomic Elements in the 22q11.2 Deletion Syndrome Microdeletion Types and Corresponding Repeats

**Figure 3:**
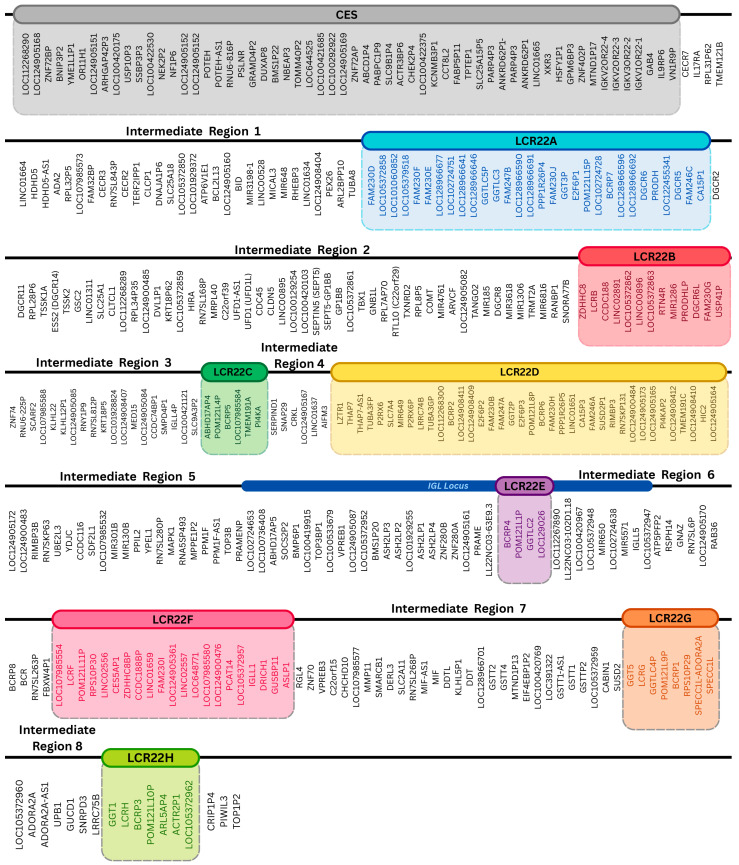
Simplified visualization of the 22q11.2 deletion syndrome segment of interest. The repeat regions are illustrated as circle tubes, and the IRs are defined by the black dotted boxes. In the figure are shown the functional genomic elements within the repeats and IRs.

**Figure 4:**
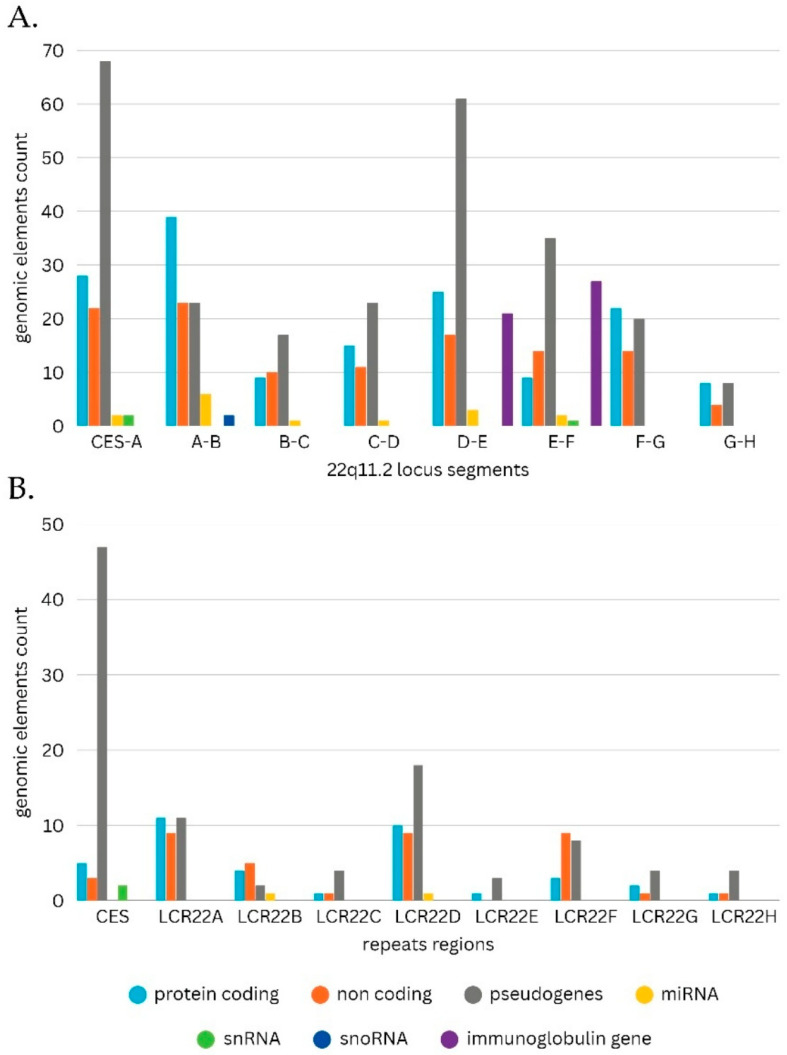
Statistical analysis of the functional genomic elements of 22q11.2 deletion syndrome. (**A**) Content analysis of all functional genomic elements contained in each of the 22q11.2 locus segments. (**B**) Content analysis of all functional genomic elements comprising the 22q11.2 repeat regions.

The results of the third part of the genomic analysis showed that the 22q11.2 deletion syndrome map extends approximately 9.5 Mb, spanning the chr22: 15,709,205–25,164,881 region, and encompasses 475 functional elements (Figure 3). The repeat regions are well defined, along with the elements that compose them, and the Immunoglobulin lambda locus (IGL) is also included (Figure 3 and Figure 4). In regard to this mapping effort, other similar arrangements of the 22q11.2 locus were studied and annotated in order to pinpoint the position of the identified repeats relative to each other and to neighboring genomic functional elements [22,81,82]. Although some inconsistences amongst publications were observed, as well as the absence of a number of elements, the bibliographical data are mostly congruent. The composition analyses revealed that the pseudogene content is high specifically in regions IR1 and IR5, protein coding genes were predominant in regions IR2 and IR7, non-coding element content was steadily average and miRNA, snRNA and snoRNA levels were steadily low or even absent (Figure 4).

Immunoglobulin genes were, expectedly, only present in segments IR5 and IR6, aligning with the location of the IGL region. Amongst the repeats, the highest pseudogene content was exhibited by the CESRR, followed by LCR22D. As for protein coding genes, they were more prevalent in LCR22A and LCR22D. Additionally, a distinct lack of snRNA, miRNA and snoRNA was observed, with only CESRR containing snRNA and only LCR22B and LCR22C containing miRNA. The 475 functional genomic elements found in the chr22: 15,709,205–25,164,881 region were categorized in 59 generalized groups, some of which include subgroups, and their prevalence in the 22q11.2 locus was studied (Supplementary Dataset S5). The groups “POM121L pseudogenes”, “BCR pseudogenes”, “Antisense RNAs” and “Long intergenic non-protein coding RNAs” occupied the highest number of the descripted segments (seven different segments), followed by “RNA, 7SL, cytoplasmic pseudogenes” (six different segments), “Zinc fingers C2H2-type and zinc finger pseudogenes”, “Solute carrier families and pseudogenes”, “Long non-coding RNAs with non-systematic symbols”, “Long non-coding RNAs with FAM root symbol”, “MicroRNAs” and “Gamma-glutamyltransferases” (five different segments). In regard to content, the group “Immunoglobulin lambda locus at 22q11.2” contained the highest number of functional elements (87 elements), followed by “Long non-coding RNAs with FAM root symbol”, “MicroRNAs” and “Antisense RNAs” (13 elements each) (Table 4). In order to determine possible functional elements of interest, a search was conducted with the aim to explore whether any of the identified groups exhibit similar characteristics to FAM230 members and BCR pseudogenes. Based on the results, the FAM230 subgroup spans four segments and includes eight elements, while BCRPs occupy seven segments and include eight pseudogenes.

Regarding their location, both can be found almost exclusively within LCRs, with the only exception being BCRP8, found in IR6, although its position is arguably very proximate to the bounds of LCR22F. Similar parameters (spanning 4–7 or more segments) are exhibited by the groups “Zinc fingers C2H2-type and zinc finger pseudogenes” (6 elements), “Solute carrier families and pseudogenes” (7 elements), “Long non-coding RNAs with non-systematic symbols” (6 elements), “Long non-coding RNAs with FAM root symbol” (13 elements), “RNA, 7SL, cytoplasmic pseudogenes” (7 elements), “MicroRNAs” (13 elements), “Gamma-glutamyltransferases” (8 elements), “POM121L pseudogenes” (7 elements), “coiled-coil domain containing and pseudogenes” (4 elements), “CD molecules” (4 elements), “Antisense RNAs” (8 elements) and “Long intergenic non-protein coding RNAs” (13 elements) (Table 4). Interestingly, the groups “Gamma-glutamyltransferases” and “POM121L pseudogenes” only occupy LCR regions, while “RNA, 7SL, cytoplasmic pseudogenes” elements are positioned exclusively in IRs.

The remaining groups’ locations relative to the 22q11.2 segments involve both IR and repeat regions and are therefore of unclear importance regarding their role in deletion events. Furthermore, a more targeted approach was employed to investigate elements that are exclusively found within LCR22A and LCR22D (Table 5). As a result, three groups were identified, namely “PPP1R26 pseudogenes” (two elements), “E2F6 pseudogenes” (three elements) and “Carbonic anhydrases” (two elements). Notably, the subgroups “Family with sequence similarity 246” and “Family with sequence similarity 247” also occur only in LCR22A and LCR22D. Each of these groups underwent evaluation to deduce the likelihood of their involvement in NAHR events for the A-D microdeletion. It should provide a concise and precise description of the experimental results, their interpretation, as well as the experimental conclusions that can be drawn.

### 3.6. Candidate break points in 22q11.2 Genetic Locus

The PATRR analysis of the nine repeat regions (Figure 1 and Figure 3) identifies ten palindromic AT-rich repeats (Table 6). In LCR22A, five PATRR regions, consisting of repeats 10 to 30 bases in size, were found located in the following positions: (1) chr22: 18,873,531–18,886,016 (485 PATRRs), (2) chr22: 18,901,877–18,915,150 (275 PATRRs), (3) chr22: 19,066,841–19,075,802 (550 PATRRs), (4) chr22: 19,099,947–19,111,917 (558 PATRRs) and (5) chr22: 19,265,463–19,271,486 (513 PATRRs). In LCR22B, a single PATRR region was observed, consisting of repeats 9 to 30 bases in size located in the chr22: 20,716,912–20,722,958 (594 PATRRs) position. In LCR22D, two PATRRs regions were detected consisting of repeats 8 to 30 bases in size, in positions (1) chr22: 21,570,774–21,577,395 (429 PATRRs) and (2) chr22: 21,730,713–21,744,287 (934 PATRRs). In LCR22F, one PATRR region, consisting of repeats 7 to 30 bases in size, was found located in chr22: 23,793,283–23,798,219 (156 PATRRs). Finally, in LCR22H, one PATRR region, consisting of repeats 6 to 30 bases in size, was detected positioned in chr22: 25,066,306–25,067,585 (371 PATRRs). The above identified palindromic AT-rich repeats are possibly a contributing factor to the 22q11.2 chromosomal rearrangements, as they are present in five out of nine repeat regions, including LCR22A and LCR22D, which participate in the most frequent deletion subtype, the A-D deletion.

Additional factors likely implicated in deletion events were considered for this study, upon examination of the 18 groups of interest that exhibited traits similar to FAM230 and BCRPs or resided solely within LCR22A and LCR22D. These include 13 groups with similarity to FAM230 and BCRPs and 5 groups located in LCR22A and LCR22D [22,53,83,84].

**Figure 5:**
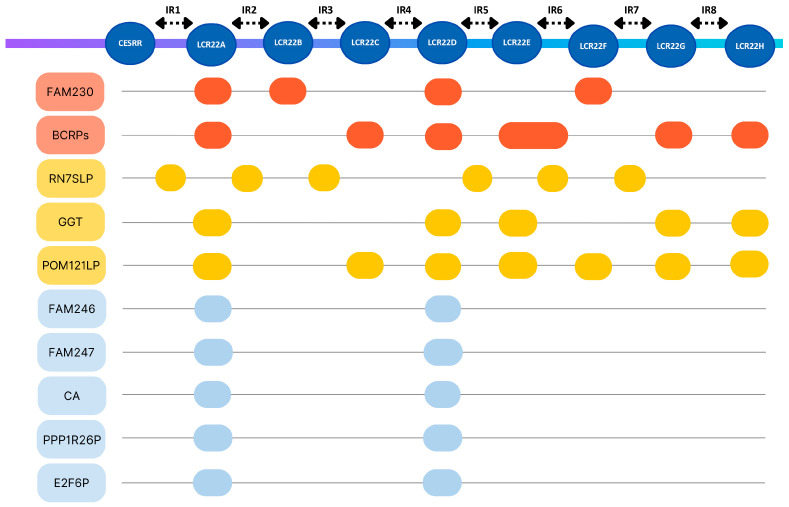
The selected segments of interest and the candidate breakpoint groups. In orange color are shown the FAM230 and BCRPs, in yellow the groups forming patterns similar to FAM230 and BCRPs, and in blue the groups occupying exclusively LCR22A and LCR22D.

After the evaluation of their relative positions both to the 22q11.2 deletion syndrome segments of interest, eight groups were distinguished for their potential as candidate breakpoints. These groups include: (i) subgroup “Family with sequence similarity 246” (FAM246), (ii) subgroup “Family with sequence similarity 247” (FAM247), (iii) “RNA, 7SL, cytoplasmic pseudogenes” (RN7SLP), (iv) “Gamma-glutamyl transferases” (GGT), (v) “POM121 transmembrane nucleoporin like pseudogenes” (POM121LP), (vi) “Carbonic anhydrases” (CA), (vii) “PPP1R26 pseudogenes” (PPP1R26P) and (viii) “E2F6 pseudogenes” (E2F6P) [85,86]. The groups (iv) and (v) correspond exclusively to LCR22s, the groups (i), (ii), (vi), (vii) and (viii) are found solely in LCR22s A and D, and group (iii) occupies only intermediate region positions (Figure 5).

**Figure 6:**
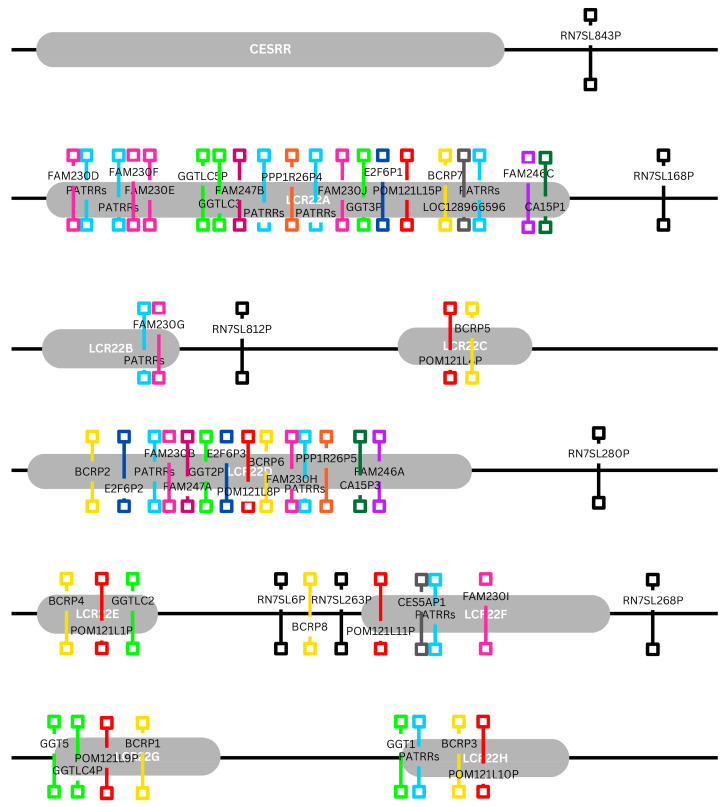
Simulation of the 22q11.2 locus segment of interest with the major identified PATRRs. In different colors is shown the twelve major groups of candidate breakpoints.

Four distinct patterns were observed in the identified PATRRs at the segments of interest within the 22q11.2 deletion syndrome genetic locus after the categorization of each group’s elements (Figure 6). The first pattern consisted of FAM230 members containing PATRRs, with FAM230I being the only exception. It should be noted that not all PATRRs were located in FAM230 members, but were also in three other functional elements: LOC128966596 (LCR22A), CES5AP1 (LCR22F) and GGT1 (LCR22H) [87]. The second recurrent configuration was GGT-POM121LP-BCRP. This pattern is subject to change amongst repeat regions, as observed in LCR22B and LCR22H, where the GGT locus is absent and in LCR22A, and in LCR22D the configuration is enriched with the additions of FAM247 members, PPP1R26 and E2F6 pseudogenes. The third pattern concerns LCR22A and LCR22D and involves the pairing of a FAM246 member and a carbonic anhydrases pseudogene (FAM246-CA). The fourth and final pattern involves the flanking of almost all repeat regions by an RNA, 7SL, cytoplasmic pseudogene.

## 4. Discussion

### 4.1. The Relationship Between the 22q11.2 Deletion Size and the Clinical Phenotypic Spectrum

The phenotypic impact of the size of the 22q11.2 microdeletion has been previously investigated, and, although no clear correlation between a given subtype and associated disease has been found, there have been differentiations noted in regard to the location of the deleted segment. More specifically, the deletion subtypes A-C, B-D, and C-D have been found to result in milder phenotypes, while distal deletions (22q11.2 distal deletion syndrome) cause phenotypes that diverge from the typical clinical spectrum of DiGeorge Syndrome. In this study, it has been found that most of the syndrome phenotypes correspond to the proximal segments of the 22q11.2 locus (IR1–IR5), while the distal segments were less phenotypically dense. However, no direct correlation between deletion type and clinical diagnosis was uncovered, which could imply the involvement of epigenetic factors or modifiers. Rozas, Benavides and León et al. 2019 reached similar conclusions when studying the proximal deletion subtypes, aiming to uncover a connection between deletion size and congenital heart and palate anomalies [16]. They proposed that the genes responsible for these manifestations reside in the A-B (IR2) region in order to explain the lack of a significant association between the deletion size and penetrance. However, according to the case study results, congenital heart and palatal defects are present even when the A-B region remains intact.

Primary immunodeficiency and 22q11.2 deletion syndrome are fundamentally linked through the development of the thymus gland [17,88]. This connection occurs because the genetic microdeletions on chromosome 22 disrupt the embryonic development of the thymus, the organ where T-cells mature [88]. In the majority of reported cases, the thymus is underdeveloped (hypoplastic), resulting in lower T-cell counts that may cause frequent respiratory or ear infections, but these often decrease as the child grows [19]. However, in approximately 1% of cases, the thymus is entirely absent (aplastic) [19]. This results in a life-threatening lack of T-cells, creating a condition similar to Severe Combined Immunodeficiency (SCID) that requires urgent treatment, such as a thymus transplant, to survive. Beyond T-cell issues, problematic biological pathways associated with 22q11.2 deletion syndrome can also affect B-cell function and antibody production, further complicating the body’s ability to respond to vaccines and increasing the risk of autoimmune disorders where the immune system mistakenly attacks its own tissues [21,89].

### 4.2. The Significance of Candidate Breakpoints in the Study of Genetic Disorders

In this study, eight functional genomic element groups, which possibly form four distinct patterns, are proposed as candidate breakpoints. These groups include FAM246, FAM247, RN7SLP, GGT, POM121LP, CA, PPP1R26P and E2F6P, which could participate in 22q11.2 deletion events, along with the ten PATRRs regions that were identified in five out of nine repeat regions of the 22q11.2 locus. The above identified breakpoints require further study to assess their potential role in 22q11.2 deletion syndrome as well as other genetic conditions. Only in recent years have breakpoints been included within the scope of chromosomal rearrangement studies, and an ultimate definition of their characteristics and structures has yet to be established. PATRRs are distinctive sequences rich in AT repeats that tend to form unstable structures and are frequently located near breakpoints. Both breakpoints and PATRRs are recognized for their roles in chromosomal rearrangement events. Since DNA breakpoints serve as hotspots for chromosomal rearrangements, such as inversions, translocations and deletions, they can lead to the emergence of various chromosomal disorders. The current study’s results could contribute to the furtherance of knowledge regarding DNA breakpoints and their features, which could be utilized in the study of various genetic disorders whose emergence is rooted in such elements, while also contributing to DNA-editing research. Uncovering the mechanisms which influence chromosomal mutations will improve the understanding of the genotype–phenotype relationship and ultimately optimize the medical care of affected individuals from a pharmacogenetic perspective.

### 4.3. Limitations of This Study

The current study faced several limitations regarding the available genomic and phenotypic data on 22q11.2 deletion syndrome. Firstly, the bibliography pertaining to the distal LCRs (LCR22E-LCR22H) and the CES region of interest was insufficient and did not describe their features, position or genomic elements in detail. Therefore, the verification of the study’s findings was difficult or impossible. Secondly, it should be acknowledged that the sample size of the case study individuals (66) is small, even more so for each atypical deletion subtype. Therefore, the results and conclusions of this article are of an exploratory nature and serve to illustrate rather than represent the phenotypic consequences and genomic mechanisms of 22q11.2 deletion syndrome. Thirdly, during our research, information regarding the genetic locus of the 22q11.2 LCRs and each deletion type was either sparce or inconsistent; therefore, we were unable to establish an accurate map of the 22q11.2 region genomic elements based on bibliographical sources alone. Therefore, genomic analysis was performed to identify these regions, and the extracted results were compared with other results based on the available literature. It is also important to note that LCRs are characterized by high levels of structural variation, which further contribute to haplotype heterogeneity. Consequently, a re-mapping effort was deemed necessary to establish a baseline for our research.

Since the study of candidate breakpoints has been under consideration in recent years, there is not enough information regarding their structures, their role in recombination events, and their applicability as new pharmaceutical targets. The results of this work regarding the new candidate breakpoints are presented for the first time, and we hope that they will contribute significantly to a better understanding of these complex biological mechanisms. The association of the different types of microdeletions in 22q11.2 deletion syndrome with the clinical phenotype and biological mechanisms was not sufficient, since there was not a detailed mapping of the syndrome based on the identified types. The results of this study will be the precursor for a more accurate recording of the distinct microdeletion types and their correct correlation with the corresponding clinical phenotype.

## 5. Conclusions

The present study examines 22q11.2 deletion syndrome, with the aim to examine the genotype–phenotype relationship and uncover the structures responsible for deletion events in the 22q11.2 locus. Through this effort, nine repeat regions were determined, one of which includes a novel locus within the CES region that has not been previously mapped. Ten candidate breakpoint groups were identified, eight of which have not been previously considered as elements of interest in 22q11.2 deletion events. Additionally, five of the ten palindromic AT-rich repeats that were determined have not been previously reported and are suggested for further investigation. Finally, our results verify the phenotypic distinction between proximal and distal microdeletions. These findings require further examination as they could increase our understanding of how similar genetic disorder occur, as well as enhance individualized medical care.

## Figures and Tables

**Table 1:** The case study microdeletion types and their features. The eleven identified microdeletion types described in 66 22q11.2 deletion syndrome cases.

Microdeletion Type	Estimated Genetic Locus (T2T-CHM 13v2.0)	Estimated Length (Kb)	Based on Literature (Kb)
CES-B	15,709,205–20,781,953	5072.7	-
A-E	18,828,186–23,078,183	4250.0	-
A-D	18,828,186–21,946,658	3118.5	3000.0
A-D *	-	-	-
A-C	18,828,186–21,146,982	2318.8	2000.0
A-B	18,828,186–20,781,953	1953.8	1500.0
D-E	21,389,245–23,078,183	1688.9	-
B-D	20,520,047–21,946,658	1426.6	1500.0
C-D	21,075,991–21,946,658	870.7	1000.0
DGCR8	20,459,152–20,490,922	31.8	-
TOP3B	22,370,556–22,396,320	25.8	

* The exception of the A-D+ microdeletion type due to lack of data pertaining to its coordinates.

**Table 2:** The nine identified repeats of 22q11.2 deletion syndrome and their features.

Repeats	Based on Literature (T2T-CHM13v2.0)	Estimated Locus	Estimated Region Length (Kb)	Length Deviation			
				Deviation in Kb *		Total Repeat Region Length **	
				Start	End	Start	End
CESRR		15,709,205–17,712,271	2003.1				
LCR22A	18,828,186–19,410,796	18,844,932–19,410,915	582.6	+16.74	+0.12	+2.87%	+0.02%
LCR22B	20,520,047–20,781,953	20,519,685–20,794,637	261.9	−0.36	+12.68	−0.14%	+4.84%
LCR22C	21,075,991–21,146,982	21,075,964–21,146,878	71.0	−0.03	−0.10	−0.04%	−0.15%
LCR22D	21,389,245–21,946,658	21,417,483–21,976,644	557.4	+28.24	+29.99	+5.07%	+5.38%
LCR22E		23,043,216–23,078,183	35.0				
LCR22F		23,728,622–24,126,360	397.7				
LCR22G		24,698,271–24,775,331	77.1				
LCR22H		25,059,674–25,164,881	105.2				

* The deviation in size was calculated as the difference between the approximate and the referenced coordinates and was depicted as (+) for surplus and as (−) for deficit in bases. ** The deviation in size was expressed as a percentage (%) of the total length of the corresponding repeat region.

**Table 3:** Forecast of related disease based on the functional genomic elements of the corresponding 22q11.2 deletion syndrome microdeletion types.

Microdeletion	Individuals	Prognosticated Phenotypes	Prognosticated Phenotypes vs. Clinical Phenotypes of Individuals	
CES-B	1	86	0	-
A-B	3	86	4	VPI; ADHD; Seizures; TOF
A-D	51	108	10	TOF; VPI; ID/ID mild; ADHD; ASD; Schizophrenia; Hydronephrosis; Cryptorchidism; Inguinal hernia; Hypernasal speech
A-D+	1	116	2	Microcephaly; TOF
A-E	2	119	4	ADHD; VPI; Seizures; Hydronephrosis
B-D	4	107	1	Hydronephrosis
C-D	1	107	0	-
D-E	1	60	1	VPI
DGCR8	1	24	4	ADHD; ASD; Generalized hypotonia; Ear malformation
TOP3B	1	3	0	-

**Table 4:** The identified functional element groups based on the parameters of the FAM230 and BCRP groups. The table includes groups with a similar prevalence to FAM230 members and BCR pseudogenes.

Group/Subgroup Functional Genomic Elements	Segments of Interest	Total Segments	Total Elements
Zinc fingers C2H2-type and zinc finger pseudogenes	CESRR, IR3, LCR22D, IR5, IR7	5	8
Solute carrier families and pseudogenes	CERR, IR1, IR2, IR3 LCR22D, IR7	6	7
Long non-coding RNAs with non-systematic symbols	CESRR, IR1, LCR22A, IR2, LCR22F	5	6
Long non-coding RNAs with FAM root symbol	IR1, LCR22A, LCR22B, LCR22D, LCR22F	5	13
RNA, 7SL, cytoplasmic pseudogenes	IR1, IR2, IR3, IR5, IR6, IR7	6	7
MicroRNAs	IR1, IR2, LCR22B, LCR22D, IR6	5	13
Gamma-glutamyltransferases	LCR22A, LCR22D, LCR22E, LCR22G, LCR22H	5	8
POM121 transmembrane nucleoporin-like pseudogenes	LCR22A, LCR22C, LCR22D, LCR22E, LCR22F, LCR22G, LCR22H	7	7
Coiled-coil domain-containing and pseudogenes	LCR22B, IR3, IR5, LCR22F	4	4
CD molecules	IR1, IR2, IR5, LCR22F	4	4
Antisense RNAs	CESRR, IR1, IR2, LCR22D, IR5, IR7, IR8	7	8
Long intergenic non-protein coding RNAs	CESRR, IR1, IR2, LCR22B, IR4, LCR22D, LCR22F	7	13

**Table 5:** The identified functional element groups of interest that reside exclusively in LCR22A and LCR22D.

Group/Subgroup Functional Genomic Elements	Segments	Total Segments	Total Elements
Protein phosphatase 1 regulatory subunit 26 pseudogenes	LCR22A, LCR22D	2	2
E2F transcription factor 6 pseudogenes	LCR22A, LCR22D	2	3
Carbonic anhydrases	LCR22A, LCR22D	2	2
Family with sequence similarity 246	LCR22A, LCR22D	2	2
Family with sequence similarity 247	LCR22A, LCR22D	2	2

**Table 6:** The identified palindromic AT-rich repeats of the LCRs and their features. In the table below, the location, density and size of the PATRRs are listed, as well as the genomic elements they overlap.

Repeats	Identified PATRR Locus (T2T-CHM13v2.0)	PATRR Density/Total PATRR Count	PATRR Size (Bases)	Corresponding Elements
LCR22A	chr22: 18,873,531–18,886,016	485	10–30	FAM230D
	chr22: 18,901,877–18,915,150	275		FAM230F
	chr22: 19,066,841–19,075,802	550		FAM230E
	chr22: 19,099,947–19,111,917	558		FAM230E/FAM230J
	chr22: 19,265,463–19,271,486	513		LOC128966596
LCR22B	chr22: 20,716,912–20,722,958	594	9–30	FAM230G
LCR22D	chr22: 21,570,774–21,577,395	429	8–30	FAM230B
	chr22: 21,730,713–21,744,287	934		FAM230H
LCR22F	chr22: 23,793,283–23,798,219	156	7–30	CES5AP1
LCR22H	chr22: 25,066,306–25,067,585	371	6–30	GGT1

## Data Availability

All data and materials are provided in the Supplementary Materials.

## Supplementary Materials

The following supporting information can be downloaded at: https://www.mdpi.com/article/10.3390/genes17020248/s1, Figure S1: PRISMA flow diagram; Supplementary Dataset S1: The collection of publications that have been used in the present study; Supplementary Dataset S2: Microdeletion analysis results based on several molecular types reported in 22q11.2 deletion syndrome; Supplementary Dataset S3: Repeat region mapping results; Supplementary Dataset S4: Associated disease per segment of interest results; Supplementary Dataset S5: Genomic function elements per segment of in interest and major groups per segment results. This review was not yet registered. However, the PRISMA flowchart and 2020 Checklist are provided in the Supplementary Materials.

## Abbreviations

CES	Cat Eye Syndrome
CESRR	Cat Eye Syndrome Repeat Region
CGH	Comparative Genomic Hybridization
DGS	DiGeorge Syndrome
FISH	Fluorescence In Situ Hybridization
IVIG	Intravenous Immunoglobulin
IR	Intermediate Region
LCR	Low-Copy Repeats
MLPA	Multiplex Ligation-Dependent Probe Amplification
NAHR	Non-Allelic Homologous Recombination
OGM	Optical Genome Mapping
PATRRs	Palindromic AT-Rich Repeats
qPCR	Quantitative Polymerase Chain Reaction
SNP	Single Nucleotide Polymorphism
SCI	Severe Combined Immunodeficiency
VCFS	Velocardiofacial Syndrome
WES	Whole-Exome Sequencing
WGS	Whole-Genome Sequencing
