# Supplementary figures and images for: Genetic Mapping of the 22q11.2 Deletion Syndrome (DiGeorge Syndrome) Microdeletion Types Revealed Novel Candidate Breakpoints
Source: Genes (Basel). 2026 Feb 22;17(2):248. doi: 10.3390/genes17020248 (PMC12940653; doi:10.3390/genes17020248)

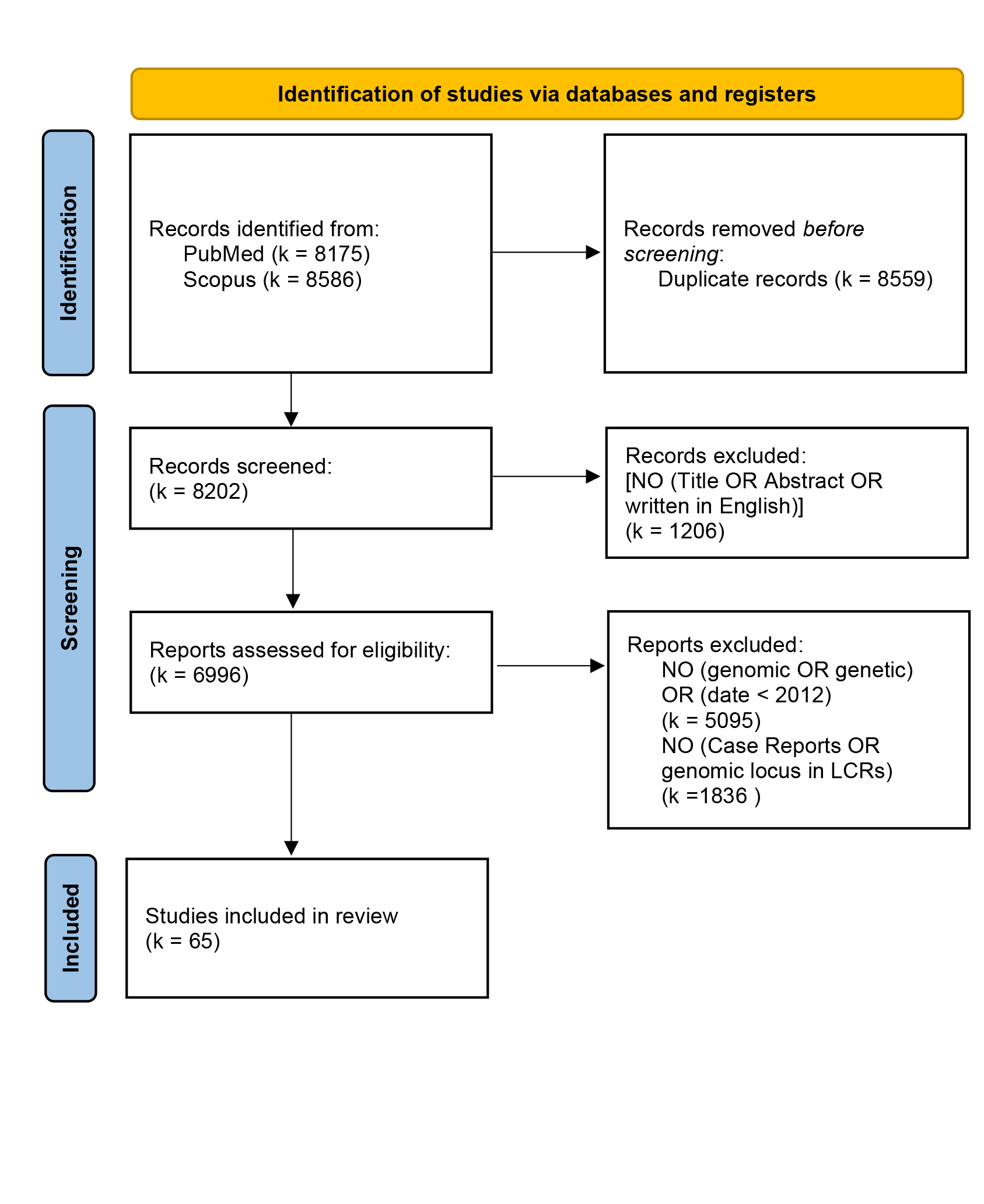

Supplement: Supplementary file 1 [file genes-17-00248-s001.zip › Supplementary Figure S1.jpg]
